# Supplementary material for: Lava dome cycles reveal rise and fall of magma column at Popocatépetl volcano
Source: Nat Commun. 2023 Jun 5;14:3254. doi: 10.1038/s41467-023-38386-9 (PMC10241900; doi:10.1038/s41467-023-38386-9)
Supplement: Supplementary file 1 — Supplementary Information [file 41467_2023_38386_MOESM1_ESM.pdf]

SUPPLEMENTARY INFORMATION

Lava dome cycles reveal rise and fall of magma column at Popocatépetl volcano

Sébastien Valade[1*], Diego Coppola[2], Robin Campion[1], Andreas Ley[3], Thomas Boulesteix[4], Noémie Taquet[4], Denis Legrand[1], Marco Laiolo[2], Thomas R. Walter[5], Servando De la Cruz-Reyna[1]

1. Universidad Nacional Autónoma de México, Instituto de Geofisica, Mexico City, Mexico
2. Università degli Studi di Torino, Dipartimento di Scienze della Terra, Torino, Italy
3. Dept. of Computer Vision & Remote Sensing, Technische Universität Berlin, Berlin
4. Volcanology Research Group, Department of Life and Earth Sciences, Instituto de Productos Naturales y Agrobiología (IPNA-CSIC), La Laguna, Spain
5. GFZ German Research Centre for Geosciences, Telegrafenberg, 14473 Potsdam, Germany

* Corresponding author (valade@igeofisica.unam.mx)

Contents of this file:

- Supplementary Figures 1-13
- Supplementary Table 1
- Caption for Supplementary Video 1
- Caption for Supplementary Data 1
- Supplementary References used in this file

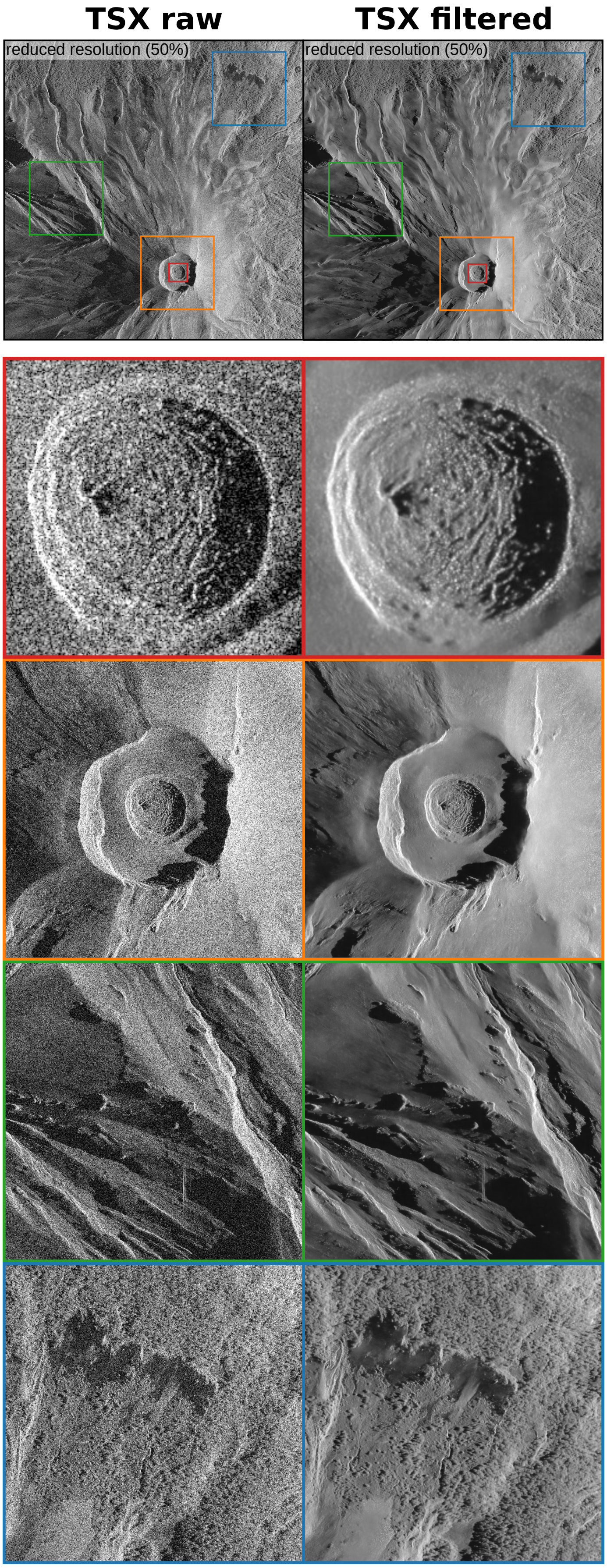

Supplementary Figure 1. Details of how the developed speckle-filter helps visualize small morphological features in TerraSAR-X images. The displayed image is acquired on 2016-05-12 on Popocatépetl volcano, i.e. a region not used during training (which exclusively used images from Colima volcano), therefore demonstrating the filter's ability to generalize.

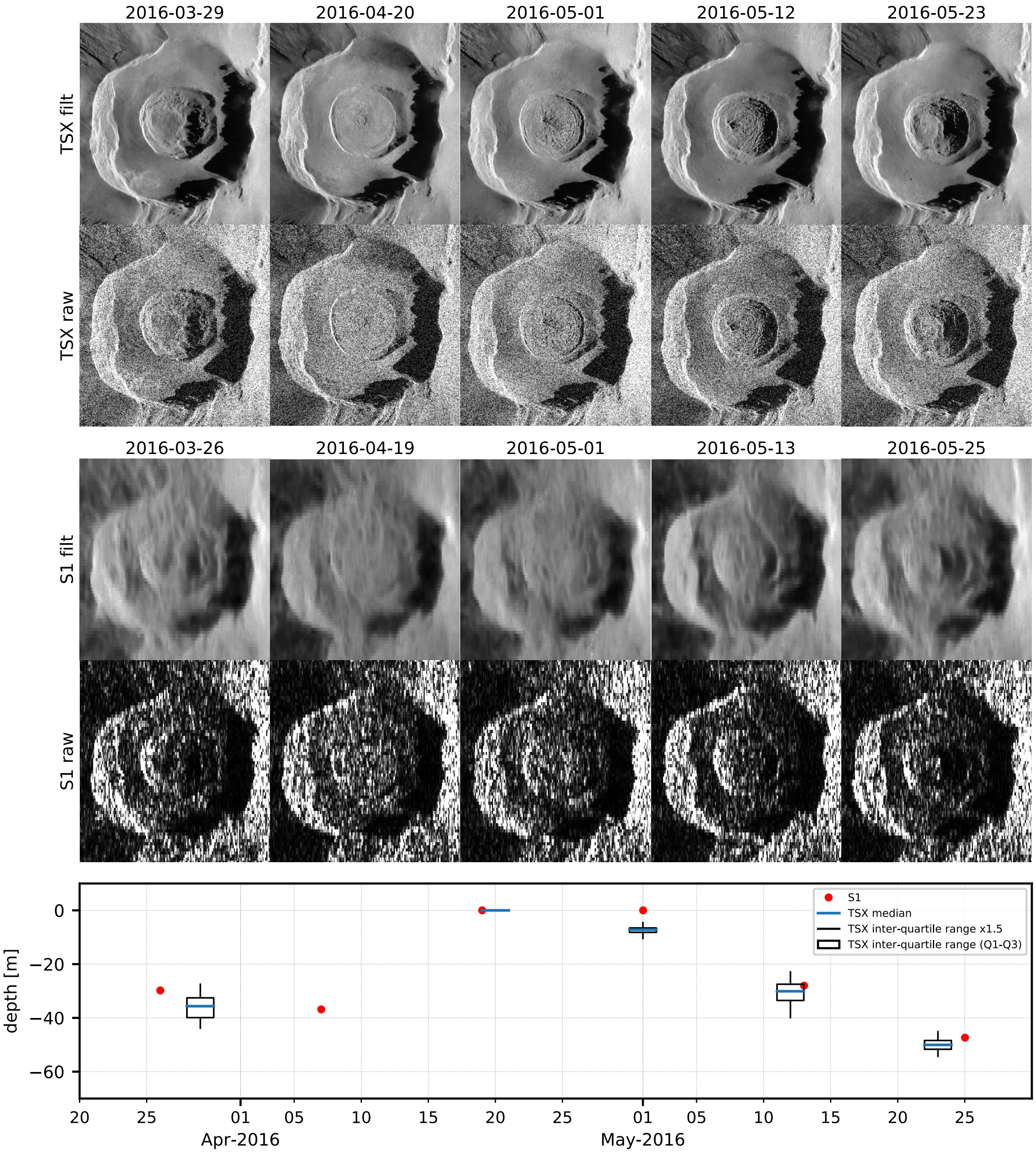

Supplementary Figure 2. Comparison between TerraSAR-X (TSX) and Sentinel-1 (S1) acquisitions, of both raw and speckle-filtered intensity images. TSX images were despeckled using the filter developed in this study (see Methods), whereas S1 images were despeckled using the filter described in Davis et al. 2020 (Supplementary Reference [1]). The bottom plot shows the crater depths recovered from the displayed TSX and S1 images, depicting the dome growth and destruction cycle in March-May 2016. The depths recovered from S1 images tend to be underestimated with respect to TSX images, likely due to a lower resolution in the range direction (2.3 m pixel range spacing for S1 IW images, against 0.91 m for TSX spotlight images) which make the detection of small shadow pixels more difficult. For example, on 2016-05-01 the TSX filtered image reveals a very fine shadow line (estimated crater depth = -7.1 m), whereas the S1 image acquired on the same day does not have the resolution to detect this small shadow (estimated crater depth = 0 m).

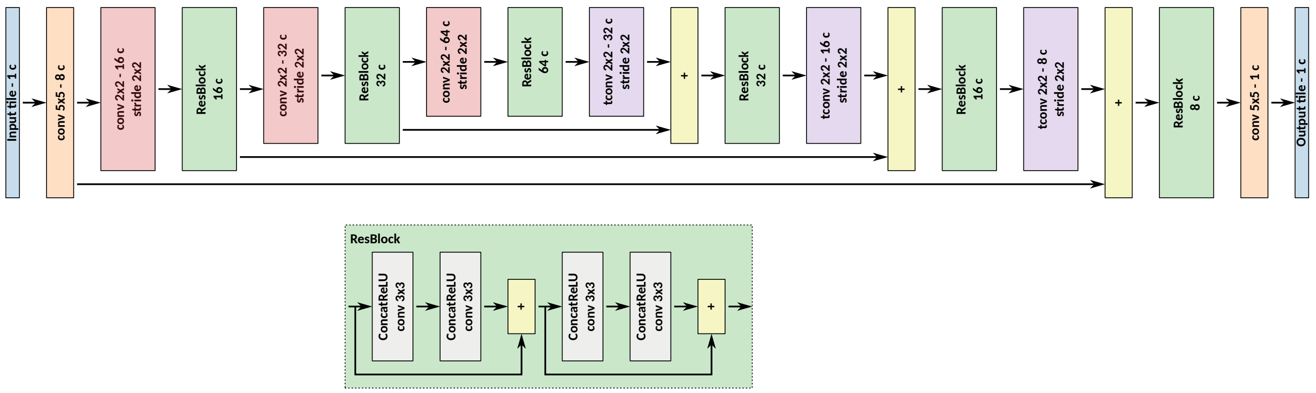

Supplementary Figure 3. Architecture of the convolutional neural network designed and trained to filter speckle from TerraSAR-X images. Input and output are single channel log intensity images. The chosen architecture follows the basic structure of a U-Net/Ladder Network with ResNet inspired processing blocks. These have forwarding bypasses resulting in improved signal and gradient flow (He et al. 2015, Supplementary Reference [2]), which is especially beneficial in a denoising scenario where the input, without noise, is to be reproduced at the output with all its details. Concatenated ReLU is used in favor of regular ReLU to prevent information loss early on in training. Strided convolutions are used for down sampling and transposed strided convolutions for upsampling. The architecture is thus a fully convolutional network which allows processing of arbitrarily sized tiles. No Dropout or BatchNorm is necessary.

Details regarding the training. Much like in Davis et al. 2020 (Supplementary Reference [1]), training is performed using pairs of image crops of the same region but with different speckle. We use L2 loss on 128x128 pixel crops. With the chosen architecture, which makes heavy use of ResNet concepts, training is stable and does not require specific tweaking.
The training data was sampled from a pool of TSX spotlight images acquired over Colima volcano (Mexico). Image pairs are selected based on the following criteria: (1) same orbit, i.e. ascending or descending, (2) at least 15 days apart, to allow for speckle to decorrelate, (3) at most one year apart, to prevent too many actual morphological changes, (4) acquired in the same or adjacent months, to force matching seasons. For these pairs, the image alignment is further refined by running Lucas Kanade alignment on 1024x1024 pixel tiles with an affine warp and an "annealing" approach of iteratively reducing a blur on the warped tile. Tile pairs with high residuals are discarded. Finally, training crops of 128x128 pixels are cropped from the reference image and paired with corresponding crops of the averaged aligned tiles, if more than 3 tiles could be aligned to that location. Note that for each reference crop the average of all corresponding crops is used as a target, rather than each corresponding crop individually. This significantly reduces the storage requirements. With the L2 loss, the network ideally converges towards approximating the average, and in this way, an implicit emphasis on pair-rich tiles is avoided.

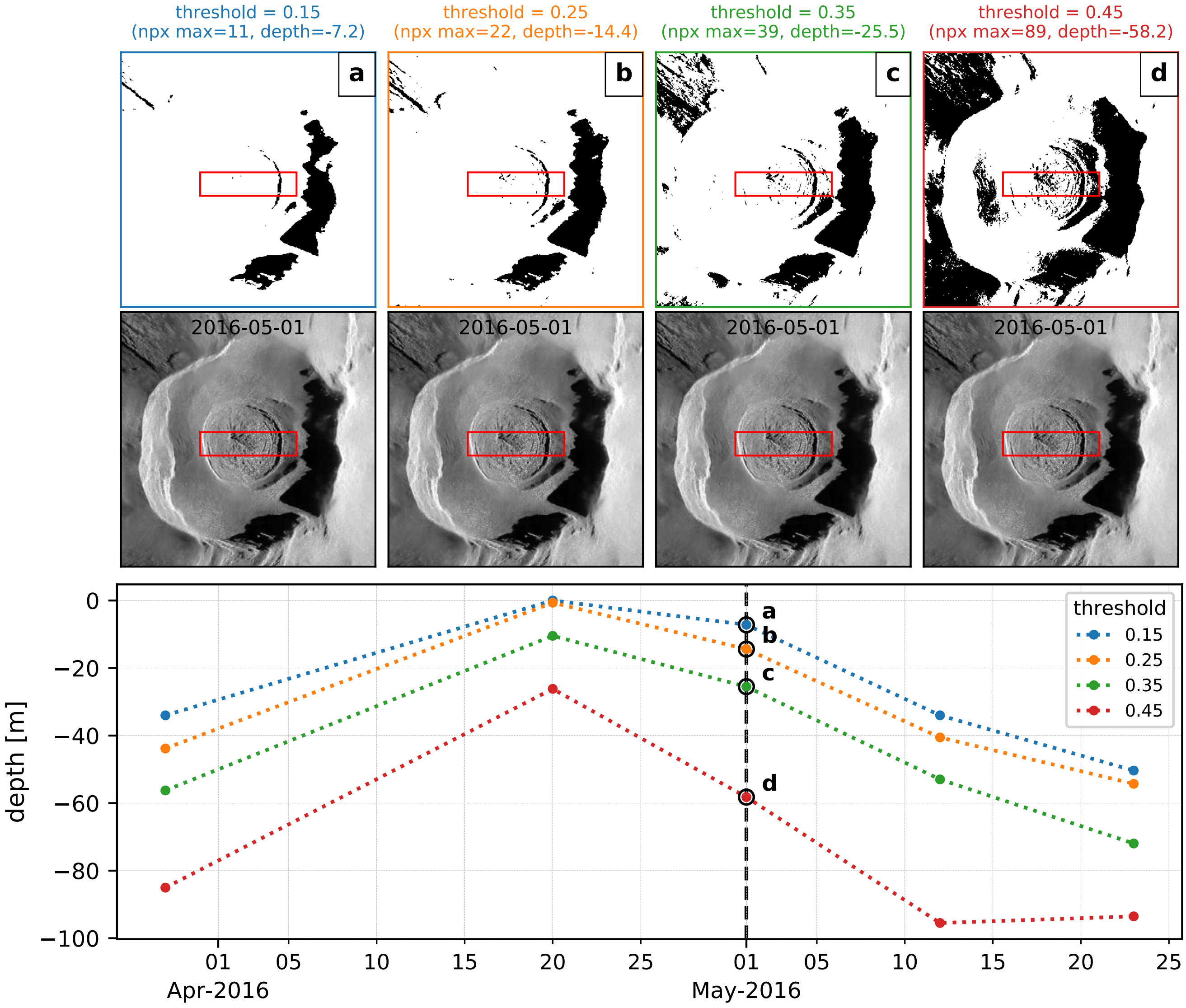

Supplementary Figure 4. Sensitivity analysis of the fixed threshold value used to segment SAR shadows in TSX intensity images. **(a-d)** Binarization of the TSX image acquired on 2016-05-01, using thresholds values of 0.15, 0.25, 0.35, 0.45, where: white = non-shadow region, and black = shadow region = region where the normalized SAR intensity is inferior to the defined threshold. The red box indicates the region where the number of pixels identified as shadow are counted to compute the crater depth (i.e., count of the maximum number of shadow pixels along a horizontal profile, as described in Methods). This region is adjusted at each SAR image to adapt to the radius of the inner-crater. The timeseries shows the inner-crater depth evolution during the dome growth-subsidence sequence of April-May 2016, calculated using the different threshold values. The values 0.35 and 0.45 clearly over estimate the SAR shadow regions, resulting in over-estimated depth values. The value 0.15 on the other hand likely under-estimates the shadow regions, as it fails to recover small shadows. Throughout this study we used a threshold value of 0.25, which was found to be the best compromise found over a representative sample of TSX images.

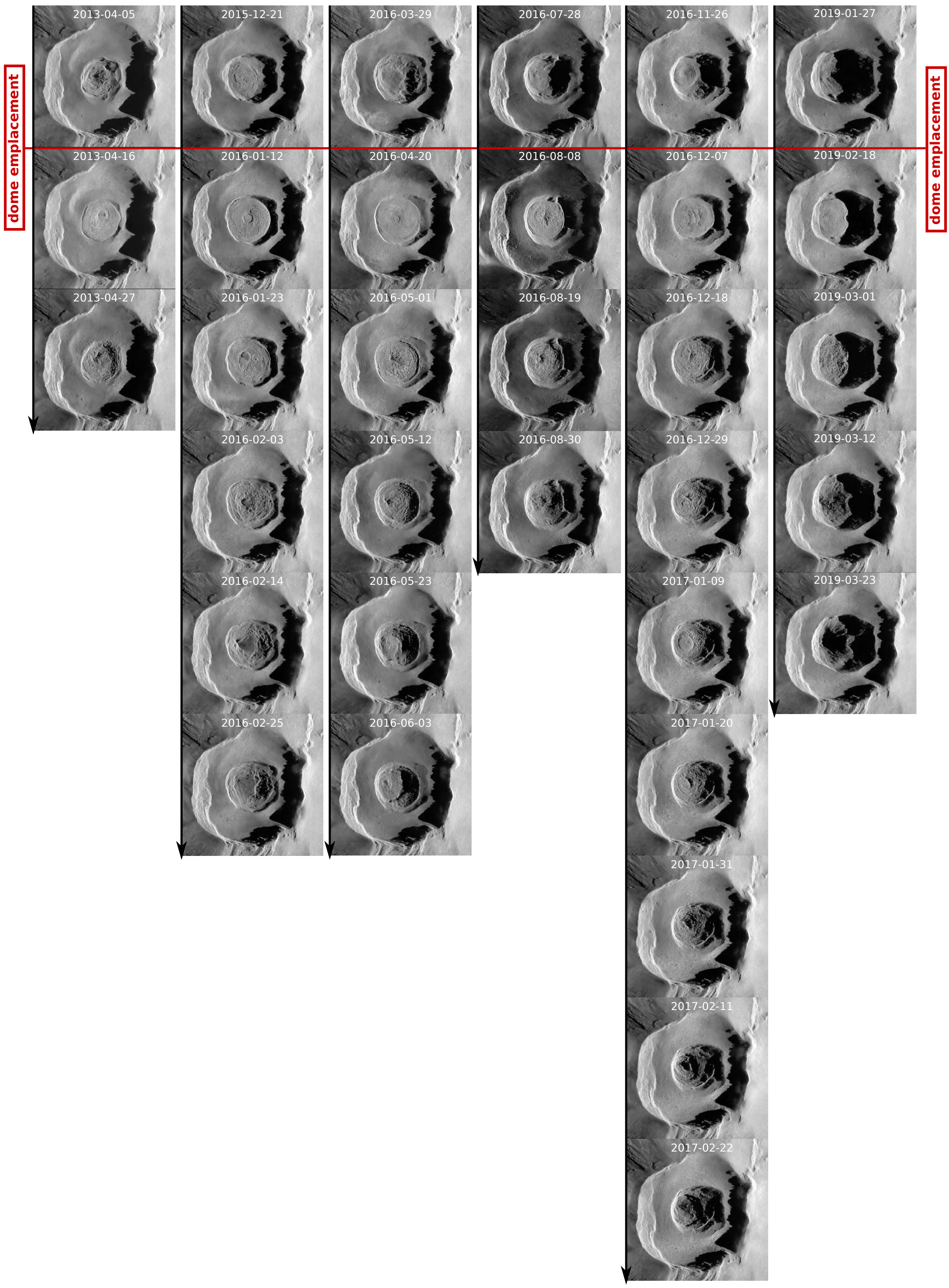

Supplementary Figure 5. Examples of dome construction-destruction cycles.

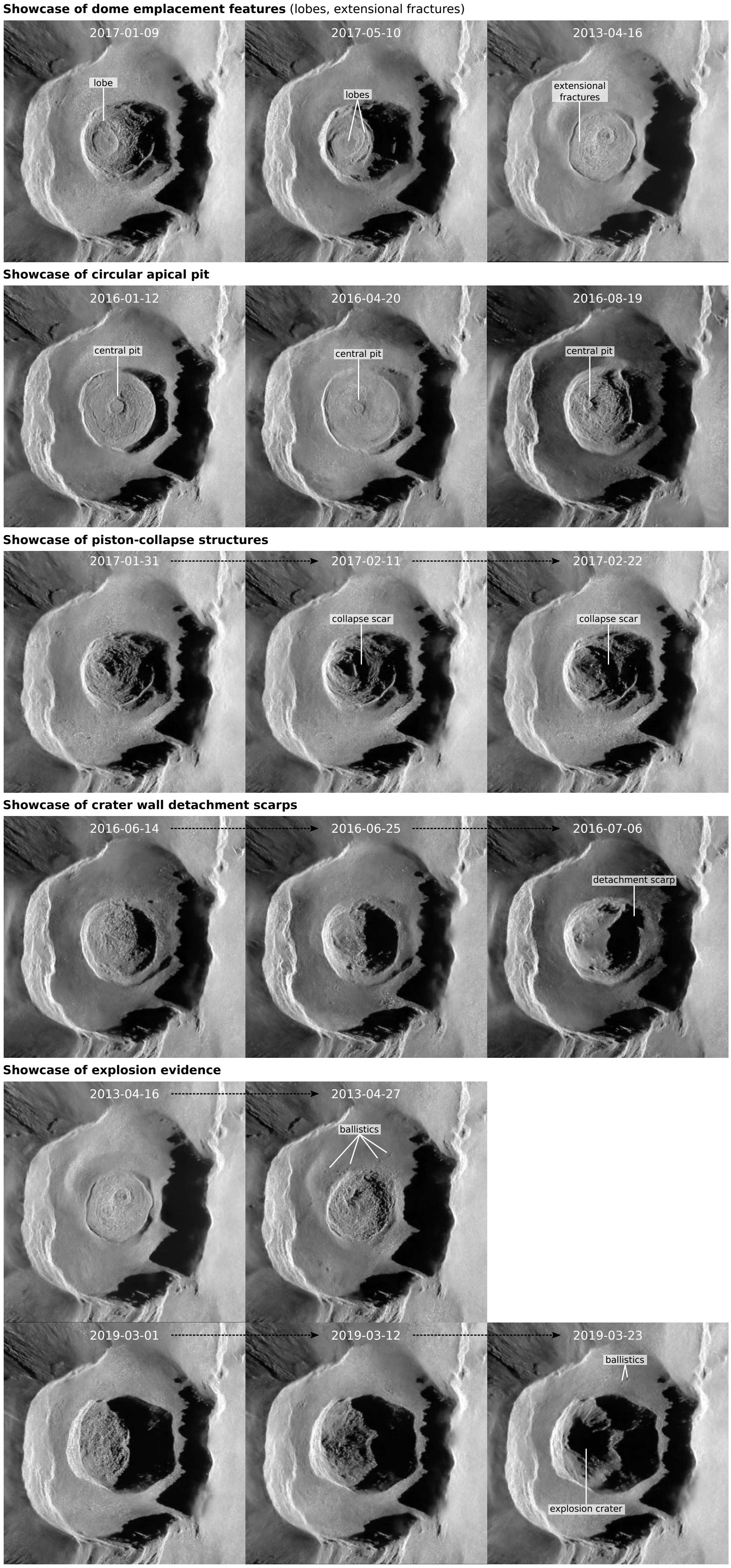

Supplementary Figure 6. Showcase of specific dome and crater morphological features.

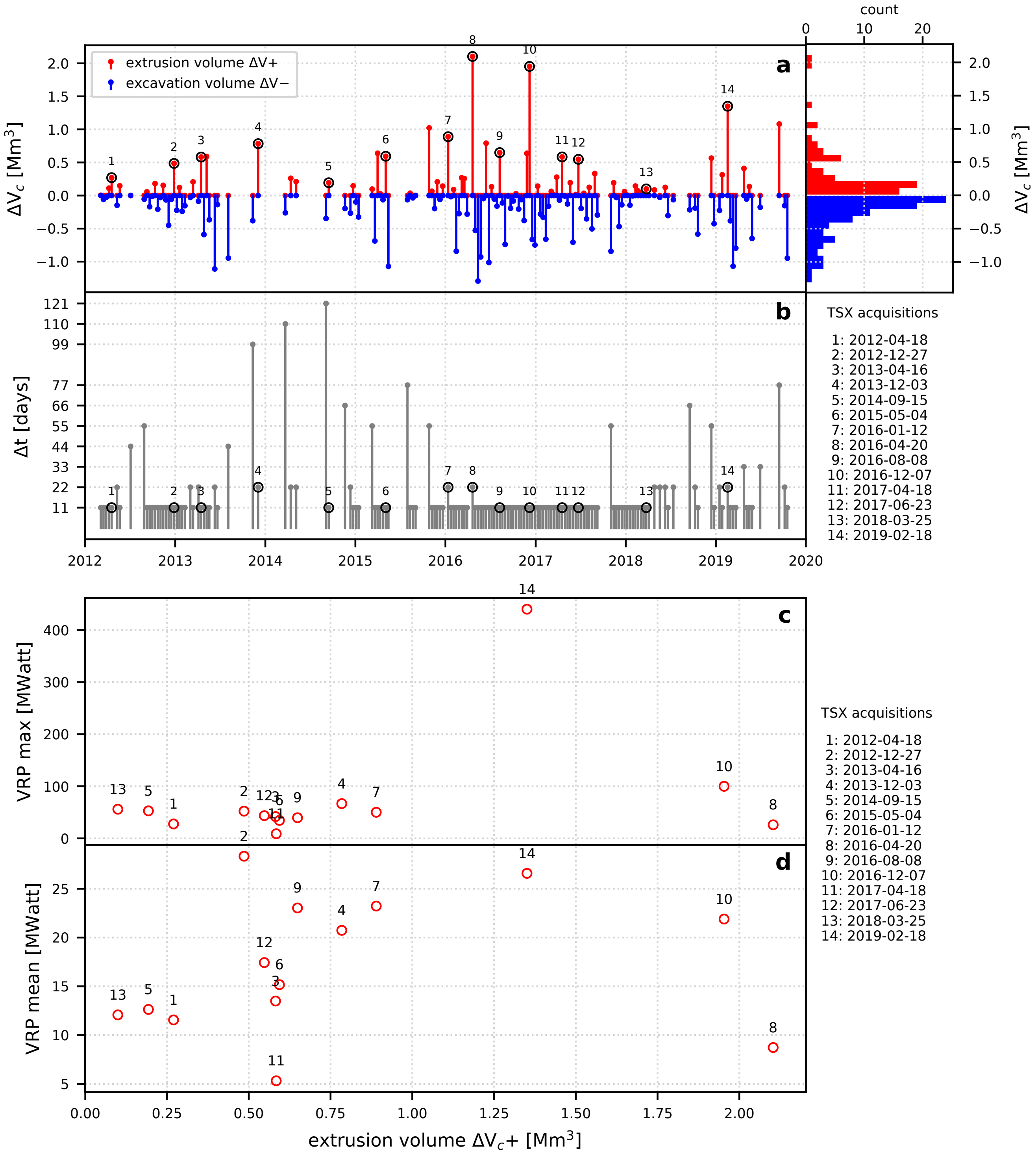

Supplementary Figure 7. Lack of clear correlation between lava extrusion volume ΔVc+ (i.e., dome volume) and thermal radiation, tested for 14 dome construction episodes (numbers 1-14, corresponding to TSX images 1-14 in Figure 2). **(a)** Crater volume change ΔVc between consecutive TSX acquisitions. **(b)** Number of days Δt separating each TSX acquisition. Extrusion volumes ΔVc+ compared to **(c)** the maximum VRP and **(d)** mean VRP values recorded during the Δt days between the two acquisitions.

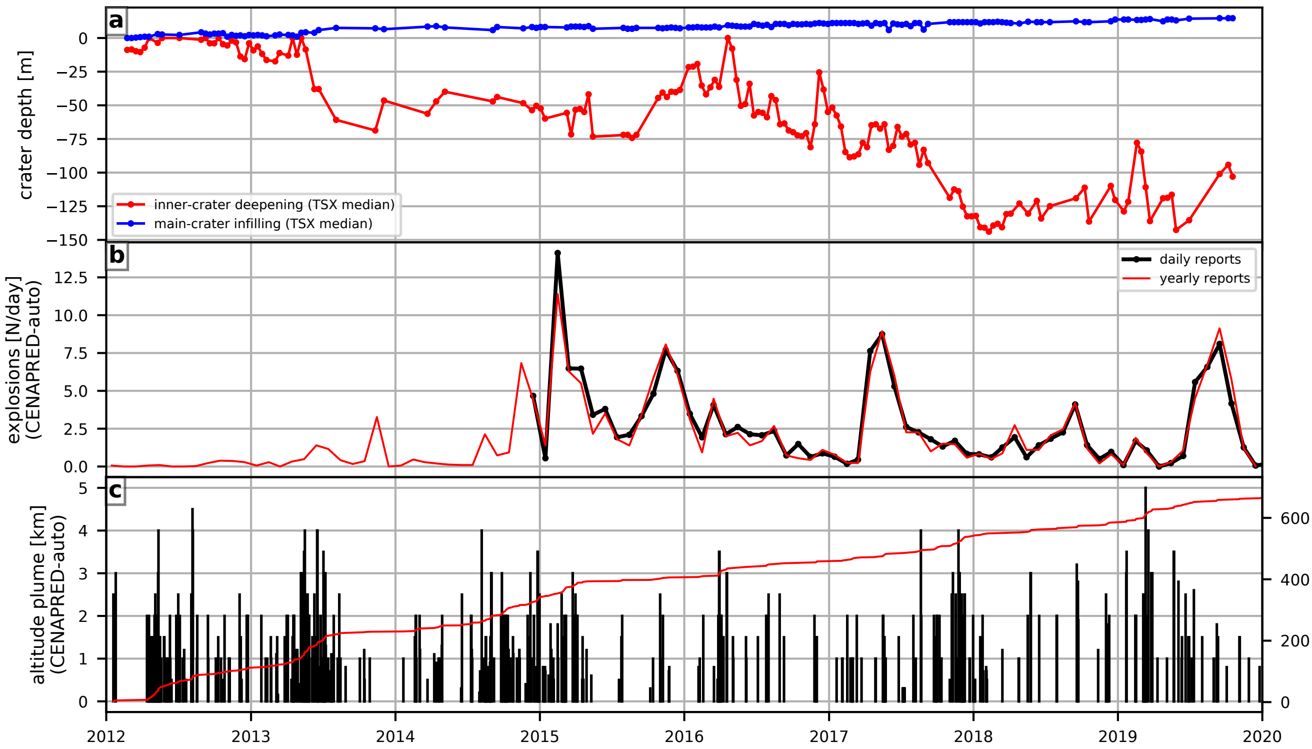

Supplementary Figure 8. (a) Inner-crater deepening (red) and main-crater infilling (blue) recovered from TSX image analysis. **(b)** Daily explosion rate and **(c)** altitude reached by ash plumes, recovered from the daily reports compiled by CENAPRED (local volcano monitoring institute in charge of surveillance of Popocatépetl), made public at: https://www.cenapred.unam.mx/reportesVolcanGobMX/

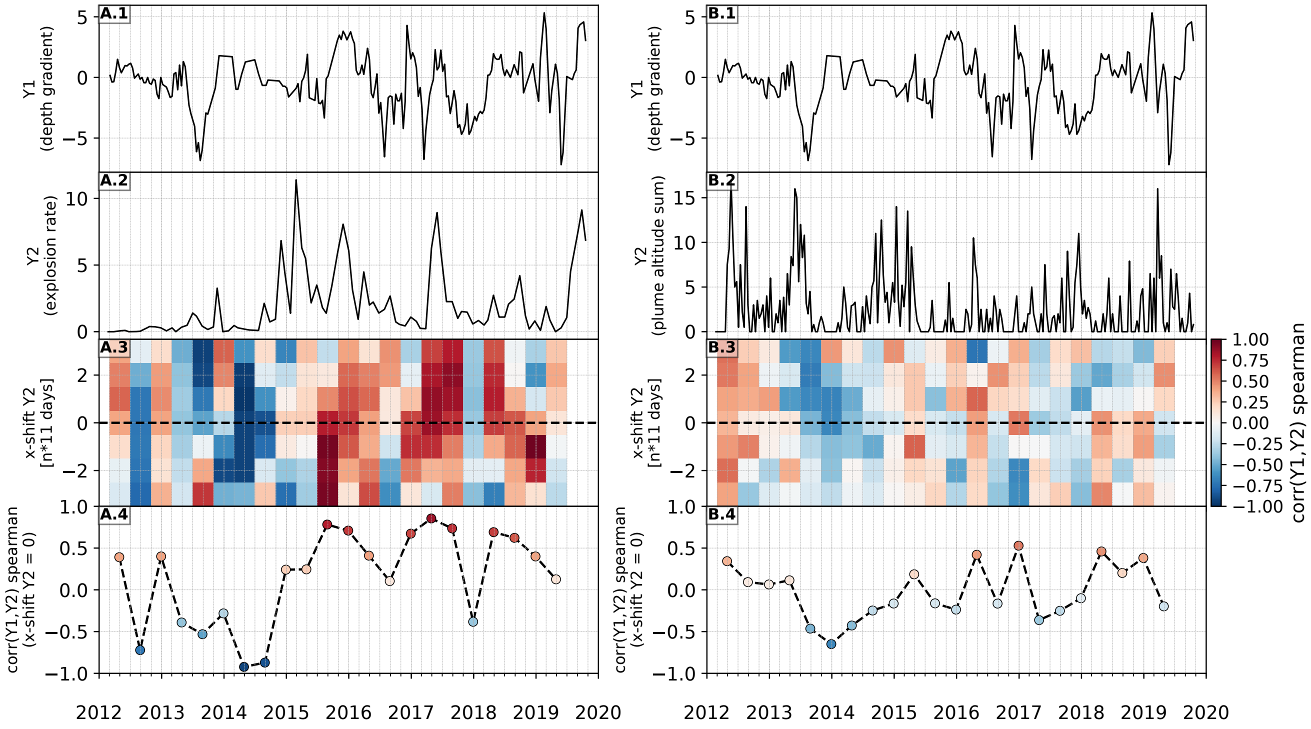

Supplementary Figure 9. Cross-correlation analysis between **(A.1-2)** crater depth gradient and explosion rate, and between **(B.1-2)** crater depth gradient and plume altitude sum (serving as a proxy to the ash emission quantities). **(A.3, B.3)** Spearman's rank correlation coefficient computed in time-windows of 4 months, and tested with a temporal x-shift ranging between +/- 3 samples (i.e., 3·11 days). **(A.4, B.4)** Spearman's correlation with x-shift = 0. Spearman correlation was chosen over the Pearson correlation because it is better suited for variables which variables non-normally distributed, with outliers, and with non-linear relationship.

Symbol	Description	Value	Unit	Source
L	length of magma column (up to mean reservoir depth)	10	km	González-Mellado & Cruz-Reyna 2008
R	volcanic conduit radius (Stevenson & Blake 1998)	[8, 13, 14]	-	$\sqrt[4]{\frac{\overline{Q}\cdot\mu_1}{\pi R_c^2\cdot\xi\cdot g\cdot\Delta\rho_{1,2}}}$
V_{r0}	volume of the reservoir at t=0	2.0	km^3	this study
R_d	dyke radius connecting reservoir to deeper source	0	m	this study
T	temperature of magma	1273	K	Witter et al. 2005
ρ_{nd}	density of parent undegassed melt	2400	kg/m^3	Witter et al. 2005
ρ_{c1}	density of degassed melt at the top of conduit	2459	kg/m^3	$\rho_{c2}+\Delta\rho_{1,2}$
ρ_{c2}	density of the undegassed melt in the conduit = ρ_{nd}	2400	kg/m^3	Witter et al. 2005
$\Delta\rho_{1,2}$	density difference between ρ_{c1} and ρ_{c2}	59	kg/m^3	Witter et al. 2005 (scenario 1)
$\rho_{m,c}$	mean density of melt in conduit (constant)	2429.5	kg/m^3	$=\gamma_{c0}\rho_{c1}+(1-\gamma_{c0})\rho_{c2}=(\rho_{c1}+\rho_{c2})/2$
μ_1	viscosity of the degassed melt in the conduit	$10^{5.3}$	Pa s	Witter et al. 2005 (scenario 1)
α	mass fraction of dissolved volatiles in parent melt	3.0	wt %	Witter et al. 2005
n_c	wt % of water exsolved in upper conduit	2.0	wt %	Witter et al. 2005 (scenario 1)
γ_{c0}	volume fraction of degassed melt in magma column at t=0	0.5	-	Girona et al. 2014
β_{c0}	volume fraction of gas in magma column at t=0	0.1	-	Girona et al. 2014
A	indicator variable (0 = constant conduit melt density)	0	-	this study
Q	DRE magma input flux $\approx$ mean degassing rate	[1, 7, 10]	m^3/s	this study
R*	empirical constant	0.6	-	Stevenson & Blake 1998
ξ	Poiseuille constant	0.064	-	Stevenson & Blake 1998
μ	effective viscosity of crust	10^{18}	Pa s	Girona et al. 2014
k	elastic modulus of crust	10^{10}	Pa	Girona et al. 2014

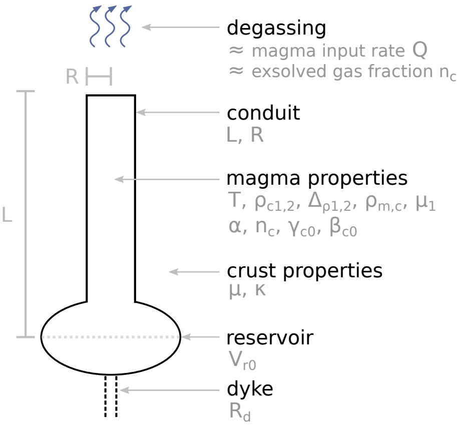

Supplementary Table 1. Parameters used to model the slow crater collapse following Girona et al. 2014 (Supplementary Reference [3]).

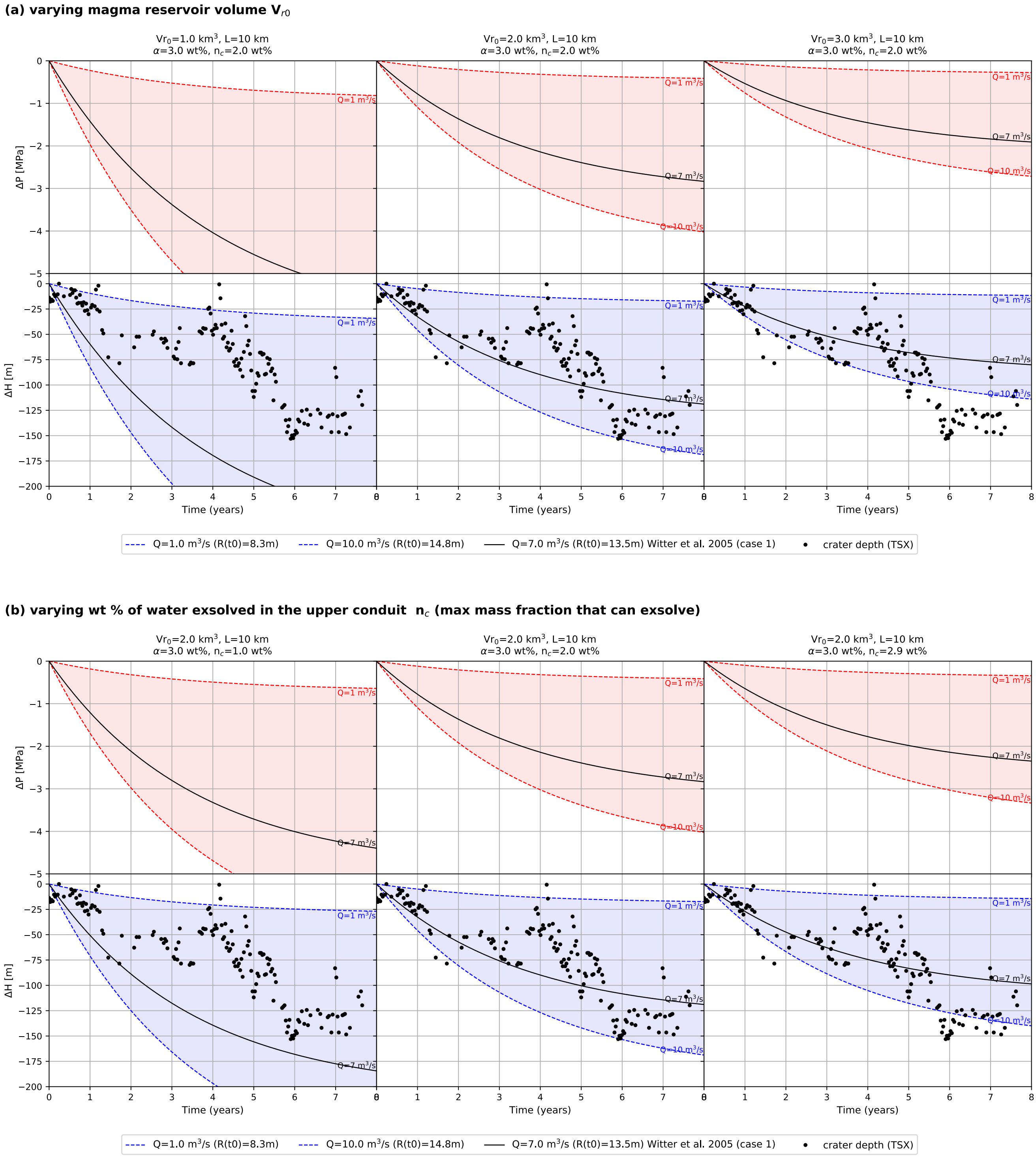

Supplementary Figure 10. Sensitivity of the Girona et al. 2014 model (Supplementary Reference [3]) to varying **(a)** initial reservoir volume V_{r0}, and **(b)** weight percentage of water exsolved in the upper conduit n_c. Values of n_c=2 wt% and n_c=2.9 wt% correspond to the two cases considered by Witter et al. 2005 (Supplementary Reference [4]) at Popocatépetl. The rest of the parameters are fixed as defined in Supplementary Table 1.

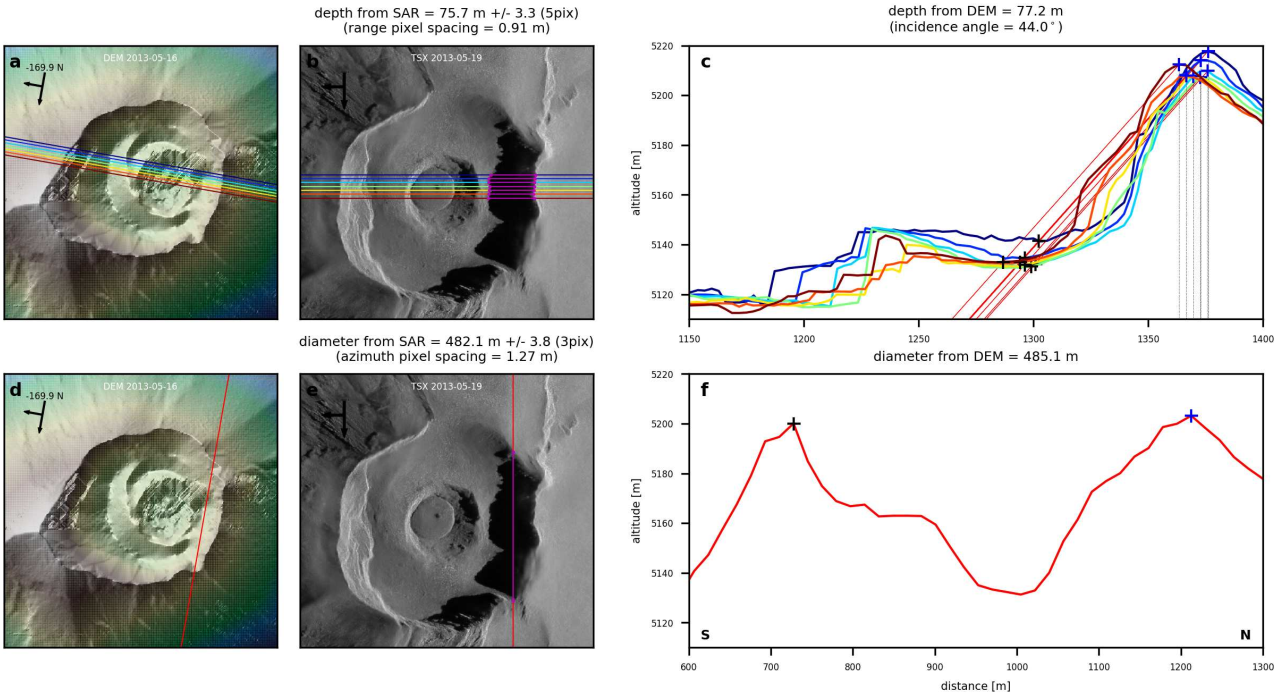

Supplementary Figure 11. Validation of the methods used to compute the crater depth (a-c) and diameter (d-f), using a Digital Elevation Model DEM (Supplementary Reference [5]) and TerraSAR-X (TSX) image acquired within a few days interval (2013-05-16 and 2013-05-19 respectively). The depth and diameter were calculated on the outer crater instead of the small inner crater in order to avoid morphological differences between the DEM and TSX acquisition.

(a) DEM overlaid with selected profiles along the "range" direction (i.e., radar line-of-sight LOS) displayed as colored lines. The satellite heading direction (-169.9°N) and look direction (right-looking) are indicated by the black arrows. **(b)** TSX image overlaid with the selected range profiles. The magenta lines indicate the detected SAR shadow at each profile. The mean depth of the crater's outer eastern wall computed from the SAR shadow (considering the SAR incidence angle = 44° from the vertical, and the pixel spacing = 0.91 m in range) is equal to 75.7 m (+/- 3.3 m, considering a +/- 5 pixels error in shadow detection), which is in very good agreement with the depth recovered from the DEM (77.2 m). **(c)** DEM cross-sections along the selected range profiles. The radar beam is drawn as red lines, starting from the crater rim (blue crosses) and intersecting the topography at the position indicated by black crosses. The mean depth of the crater's outer eastern wall computed from the DEM (i.e., altitude difference between the rim and point where the beam intersects the topography) is equal to 77.2 m. **(d)** DEM overlaid with selected profiles along the "azimuth" direction (i.e., radar flight direction). **(e)** TSX image overlaid with the selected azimuth profiles. The magenta line indicates the crater diameter along the azimuth profile. The diameter of the outer crater computed from the SAR image (considering the pixel spacing = 1.27 m in azimuth) along this profile is equal to 482.1 m (+/- 3.8 m, considering a +/- 3 pixels error in shadow detection), which is in very good agreement with the diameter recovered from the DEM (485.1 m). **(f)** DEM cross-sections along the selected azimuth profile. The crater diameter computed from the DEM along this profile (i.e., distance between the northern and southern rims, indicated by blue and black crosses respectively) is equal to 485.1 m.

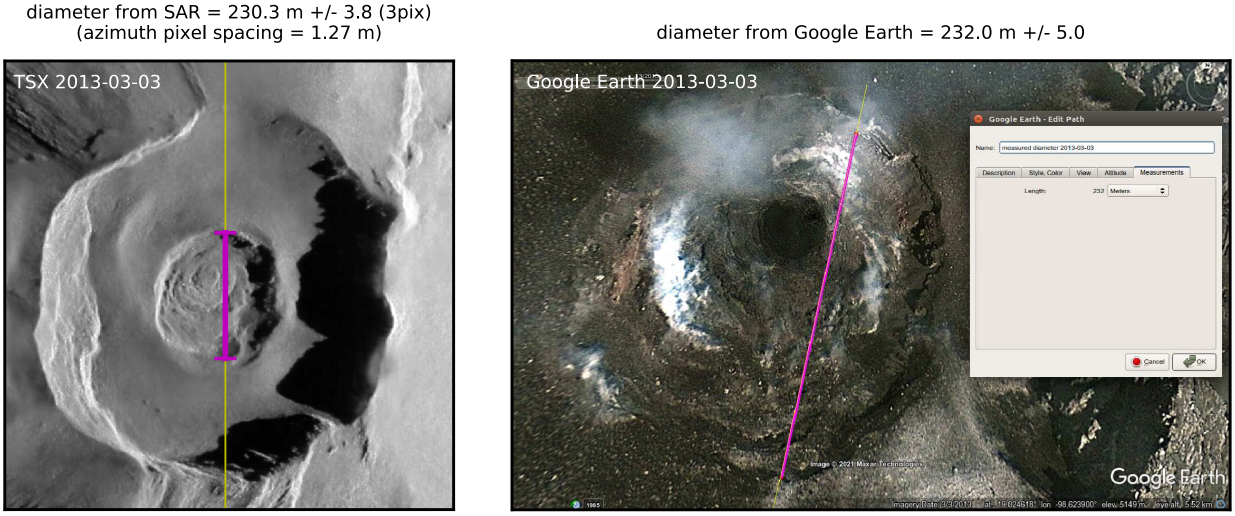

Supplementary Figure 12. Validation of the method used to compute the inner crater diameter, using a high-resolution optical image from Google Earth (Supplementary Reference [6]) and TSX image acquired on the same day (2013-03-03). The diameter automatically recovered from the SAR is equal to 230.3 m +/- 3.8, which is in good agreement with the diameter measured with Google Earth (232.0 m +/- 5). Note that because georeferencing of the TSX image is imprecise, the profile on the Google Earth image is positioned manually and therefore suffers imprecision.

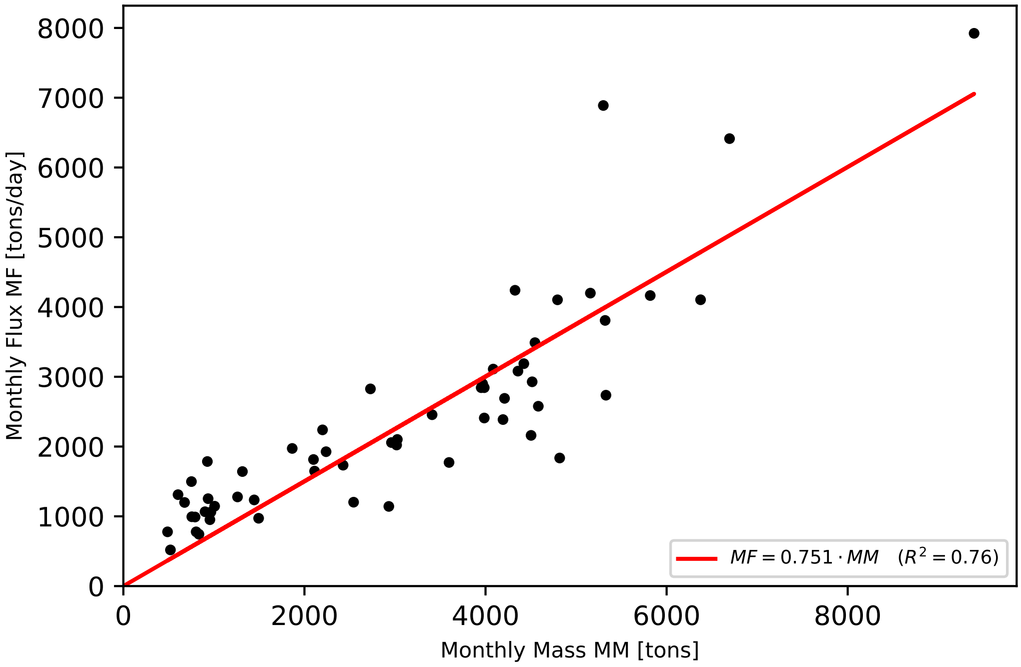

Supplementary Figure 13. Calibration of monthly SO_2 flux from OMI-derived SO_2 monthly masses at Popocatépetl.

Supplementary Video 1. Filtered TerraSAR-X intensity images acquired at Popocatépetl during ~8 years (2012-02-23 to 2019-10-18). Images are displayed in radar coordinates, where the image y-axis corresponds to the satellite motion direction, and the x-axis corresponds to the radar look direction (looking from the right). All images are acquired in descending orbit, in both spot-light (SPOT) and strip-map (STRIP) modes. Images were despeckled using the filter developped in this study (Supplementary Reference [7]), and are archived on Zenodo (Supplementary Reference [8]).

Supplementary Data 1. Source data for Fig. 2 generated in this study.

Supplementary References

1. Davis, T. et al. Reference-Free Despeckling of Synthetic-Aperture Radar Images Using a Deep Convolutional Network. *IEEE International Geoscience and Remote Sensing Symposium* (IGARSS), 3908–3911 (2020)

2. He, K., Zhang, X., Ren S. & Sun J. Deep Residual Learning for Image Recognition. 2016 *IEEE Conference on Computer Vision and Pattern Recognition* (CVPR), 770-778 (2016)

3. Girona, T., Costa, F., Newhall, C. & Taisne, B. On depressurization of volcanic magma reservoirs by passive degassing. *J. Geophys. Res. Solid Earth* 119, 8667–8687 (2014).

4. Witter, J. B., Kress, V. C. & Newhall, C. G. Volcán Popocatépetl, Mexico. Petrology, magma mixing, and immediate sources of volatiles for the 1994 - Present eruption. *J. Petrol.* 46, 2337–2366 (2005)

5. Instituto Nacional de Estadística y Geografía - INEGI. Modelo digital de elevación con resolución de 3m de la Zona del Popocatépetl, Mayo 2013 (1st ed., 2016) [model]. Aguascalientes, Aguascalientes, México. Retrieved freely from http://www.inegi.org.mx/ with no usage constraints.

6. Google Earth. Satellite image of Popocatépetl captured on 3 March 2013 by Maxar Technologies. Retrieved from Google Earth in 2022.

7. Ley, A. & Valade, S. Andreas-Ley/S2S-TSX-Colima: Version v1.0.4. (2023) doi:10.5281/zenodo.7838864 (https://zenodo.org/record/7838864).

8. Valade, S., Ley, A. & Walter, T. R. TerraSAR-X despeckled images of Popocatépetl crater (2012-2020). (2023) doi:10.5281/zenodo.7842336 (https://zenodo.org/record/7842336).
